# Radioactive Iodine Following Total Thyroidectomy Is Comparable to Lobectomy in Low/Intermediate-Risk Differentiated Thyroid Carcinoma: A Meta-Analysis

**DOI:** 10.7759/cureus.12332

**Published:** 2020-12-28

**Authors:** Ibrahim A Altedlawi Albalawi, Abdullah I Altidlawi, Hyder Mirghani

**Affiliations:** 1 Surgical Oncology, University of Tabuk, Tabuk, SAU; 2 Surgery, University of Tabuk, Tabuk, SAU; 3 Internal Medicine, University of Tabuk, Tabuk, SAU

**Keywords:** radioactive iodine, remnants ablation, lobectomy, total thyroidectomy, differentiated thyroid carcinoma

## Abstract

Radioactive iodine (RAI) is being increasingly used for remnants ablation of low/intermediate-risk differentiated thyroid carcinoma (DTC). Importantly, total thyroidectomy (TT) is in common use in the treatment of low-grade DTC to facilitate RAI despite the recommendations for lobectomy. Intermediate-risk DTC has been an arena of controversy (fueled by weighing the risks and benefits of RAI). This meta-analysis aimed to assess the role of RAI following TT in comparison to lobectomy in low/intermediate-risk patients with DTC. We identified 482 references through PubMed, Cochrane Library, EBSCO, and Google Scholar databases. The keywords used were “differentiated thyroid carcinoma”, “low/intermediate risk”, “radioactive iodine following total thyroidectomy”, “total thyroidectomy versus lobectomy and RAI”, “remnants ablation”, “recurrence”, “survival rate”, “tumor-specific cancer death”, “overall mortality”, and “tumor-specific mortality”. From the 67 full texts screened, only seven studies fulfilled the inclusion and exclusion criteria. The studies were from the USA, Australia, Asia, Mexico, and South America (63,268 patients included; five were retrospective and two prospective cohorts). No differences were found regarding recurrence and survival rate between TT followed by RAI and lobectomy alone. However, the current data were limited by the observational studies included, the pooling of both recurrence and survival rate, and the significant heterogeneity observed. The ongoing randomized controlled trials are awaited to resolve the issue.

## Introduction

The matter of radioactive iodine (RAI) in remnant ablation of differentiated thyroid carcinoma (DTC) is living and dynamic. Lin et al. in 1998 found that RAI is effective in remnant ablation following lobectomy and subtotal thyroidectomy. The authors attributed the high rate of mortality observed to the misinterpretation of follicular thyroid carcinoma as benign. Also, the unrespectable tumor was a contributing factor to mortality observed after RAI therapy [1]. In the year 2011, a higher dose of RAI was found to be more efficacious. However, clinical recurrence was not reduced [2]. A study conducted in the year 2017 in 93,530 Chinese patients showed no cancer-specific survival benefit of RAI use in intermediate-risk DTC despite the lower overall survival benefit [3]. A recent recommendation with intermediate evidence suggested integrating the patients and tumor characters on the basis of history, laboratory findings, and high-quality ultrasonography besides the patient's preference (maximalists who choose the extreme of total thyroidectomy [TT] followed by RAI to minimize recurrence versus minimalists who bear that in favor of avoiding thyroid replacement therapy if possible). Minimalists would prefer lobectomy and follow-up. The matter is complicated further by the treating team philosophy that lies in two categories: aggressive, opting for TT and RAI, and conservative, favoring lobectomy with the understanding that a completion thyroidectomy might be needed to allow for RAI use [4,5]. The preceding recommendations are excellent practices incorporating history, examination, investigation, the patients, and the local team preference. However, some areas certainly need recommendations that are more clear-cut because high-quality ultrasound might not present in a wide range of remote outreaching areas. Besides, the operator might not be a high volume. In other areas, no team is available to discuss and rather a single surgeon might take the current recommendations on his/her side and not take the patient's preference. Regional variations in RAI recommendations were observed even in developed countries [6]. In the face of the increasing recent trends towards RAI administration (despite the recommendation against its use in low-risk DTC) [7], as well as the paucity of meta-analysis on this important issue and the fact that RAI is not without complications, an update is highly needed. Thus, the study aimed to find an update regarding RAI use in patients with low/intermediate-risk DTC.

## Materials and methods

Eligibility criteria according to PICOS

Studies were included if they assessed the outcomes of RAI in patients with DTC. Studies published in English and investigating the outcomes of RAI following TT compared to lobectomy alone without RAI.

Patients

All studies on adult humans (observational and randomized studies) and comparing the effects of RAI on outcomes in patients with differentiating thyroid carcinoma were eligible. Surveys on younger age groups, those comparing outcomes in TT versus lobectomy without RAI, and those comparing the same with RAI in both arms were not included. Studies comparing RAI effects on thyroid diseases other than low/intermediate-risk DTC were not included (nodular or diffused thyroid disease, other thyroid malignancies, and high-risk DTC). Studies investigating the outcomes of RAI following lobectomy were not studied.

Outcomes measures

To be included, studies must include a comparison of TT followed by RAI with incomplete thyroidectomy without RAI in terms of mortality and tumor recurrence. We aimed to include DTC-related mortality whenever possible. However, the overall mortality was included if the above is not specified. Regarding recurrence, we found one study investigating local recurrence following RAI and fulfilling the above criteria. We did not assess for thyroglobulin, thyroglobulin antibodies, and thyroid-stimulating hormone levels [7].

Search strategy

We systematically searched PubMed (367,65 titles and abstracts, and 302 by citation and similar articles screening), Cochrane library EBSCO, and the first 100 articles on Google Scholar [8,9]. The keywords used were “differentiated thyroid carcinoma”, “low/intermediate risk”, “radioactive iodine following total thyroidectomy”, “total thyroidectomy versus lobectomy and RAI”, “remnants ablation”, “recurrence”, “tumor-specific cancer death”, “overall mortality”, and “tumor-specific mortality”. The total hits were 482, as mentioned previously, of which only seven articles fulfilled the inclusion and exclusion criteria. Furthermore, the first and second authors conducted the initial search, and the first and third authors screened the titles and abstracts for duplication removal and applying the inclusion criteria

The definition of low/intermediate risk was based on the author's reports and all the spectrum of guidelines were accepted, as it would be very difficult to come out with studies enabling a meta-analysis if we refer to specific guidelines. The widely recommended Newcastle Ottawa Scale [10] was used to assess the quality of the included studies.

Data analysis

The RevMan system for meta-analysis (https://training.cochrane.org/online-learning/core-software-cochrane-reviews/revman) was used, and the data were all dichotomous. The fixed effect was used because no significant heterogeneity was found. Funnel plots were used to assess lateralization. A p-value of <0.05 was considered significant.

**Figure 1:**
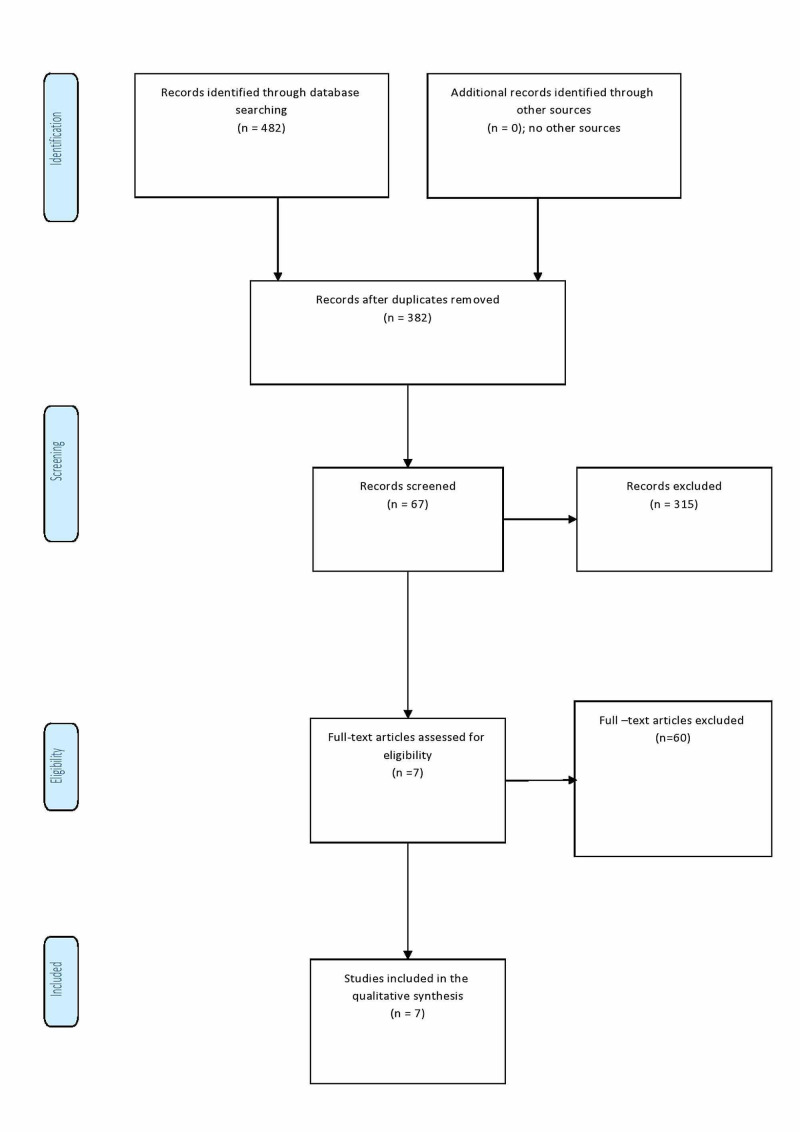
Flow diagram through the different phases of the systematic review (PRISMA flowchart). PRISMA, Preferred Reporting Items for Systematic Reviews and Meta-Analyses

## Results

**Figure 2:**
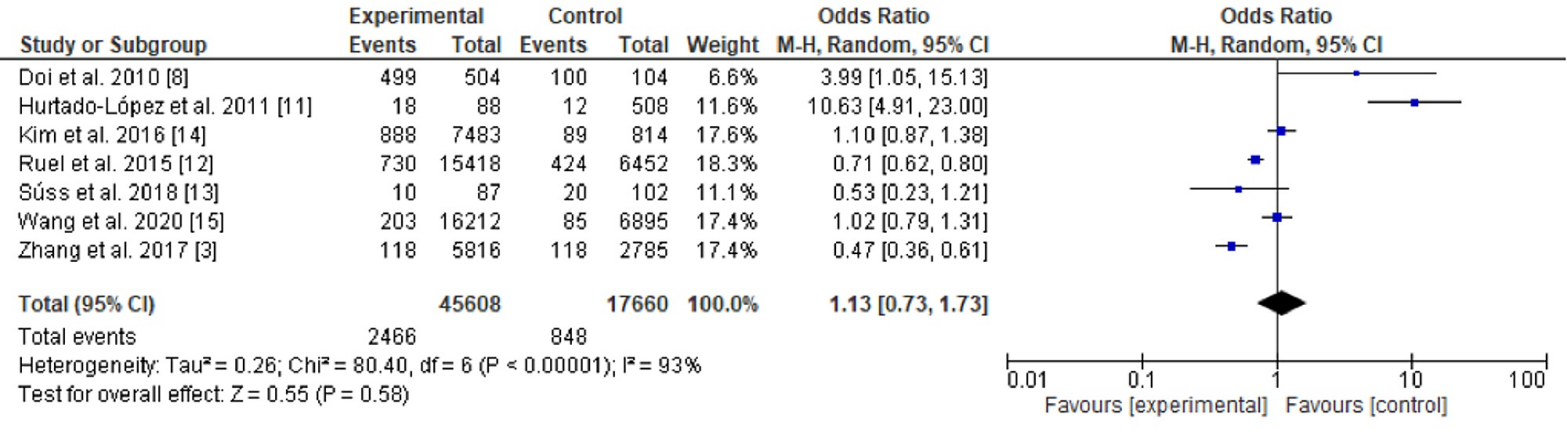
Forest plot showing a comparison of outcome measures between total thyroidectomy followed by radioactive iodine and lobectomy alone

**Figure 3:**
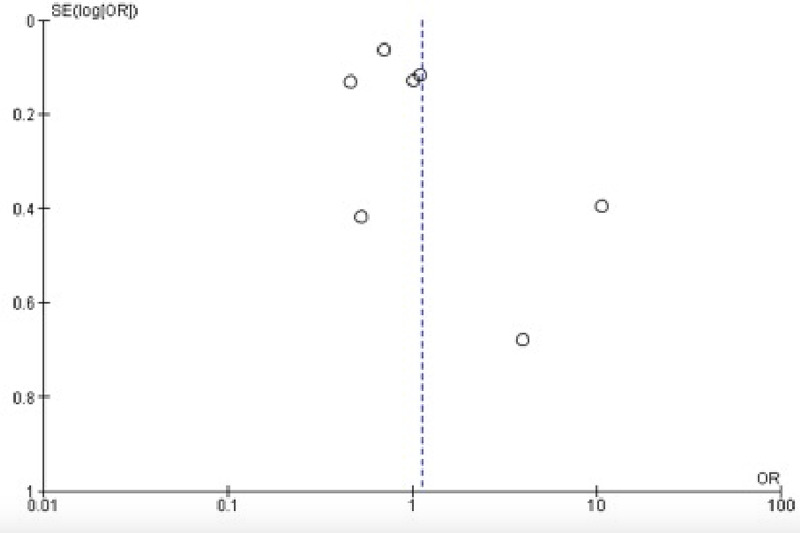
Funnel plot showing a comparison of outcome measures between total thyroidectomy followed by radioactive iodine and lobectomy alone

Out of the 482 references identified, 67 six full texts were screened, of which seven studies included in the meta-analysis (five retrospective and two prospective cohorts: three were from Asia, one from the USA, one from Australia, one from Brazil, and one from Mexico) (Table 1). The single study that assessed recurrence used three different methods (AMES [Age, Metastasis, Extracapsular tumor, Size], MACIS [Metastases, Age, Completeness of resection, Invasion, Size], and De Groot); we combined the results and reversed the outcomes because recurrence and survival are not going in the same line. Two of the included studies showed a negative effect of RAI on outcomes [8,11], three showed a beneficial effect [3,12,13], and the remaining two were neutral [14,15]. The fixed effect was used due to the substantial heterogeneity (I2=93%; p<0.001). The overall effect of RAI on survival rate was not significant (odds ratio: 1.13; 95% CI: 0.73-1.73; p = 0.58) (Figures 2, 3).

**Table 1:** Effects of radioactive iodine following total thyroidectomy effects on survival rate and recurrence among in patients with low/intermediate-risk DTC AMES: Age, Metastasis, Extracapsular tumor, Size; MACIS, Metastases, Age, Completeness of resection, Invasion, Size; TNM, tumor, nodes, metastasis; LB, lobectomy; RAI, radioactive iodine

Author	Year	Country	Methods	Patients	Control	Results	Category	p-Value
Zhang et al. [3]	2017	China	Retrospective	118/5,816	117.7/2,785	Similar survival rates	Low-risk papillary	0.164
Doi et al. [8]	2010	Australia	Retrospective	499/504	100/104	The survival rate was non-significant	Stage 1 & 2 tumor	
Hurtado-López et al. [11]	2011	Mexico	Prospective	12/508	18/88	Regional recurrence was higher among LB	Low risk using AMES, MACIS, De Groot, and TNM	0.001, 0.008, 0.025, and 0.005
Ruel et al. [12]	2015	USA	Prospective	730/15,418	424/6,452	Survival rate significant	Intermediate risk	<0.001
Súss et al. [13]	2018	Brazil	Retrospective	10/87	20/102	No benefit of 30-mCi RAI regarding outcomes	Low/intermediate risk	0.59
Kim et al. [14]	2016	South Korea	Retrospective	88.8/7,483	89/814	Survival rate not significant	Intermediate risk	0.137
Wang et al. [15]	2020	China	Retrospective	203/16,212	85/6,895	Survival rate significant	Intermediate risk	<0.001

## Discussion

There is an increasing trend toward RAI remnant ablation for the low/intermediate-risk DTC. In the current meta-analysis, TT as a primary surgery and followed by RAI and lobectomy without RAI were equal in terms of survival and recurrence rate (odds ratio: 1.13; 95% CI: 0.73-1.73; p = 0.58). Sawka et al. [16] conducted a previous meta-analysis of good quality and assessed 13 cohorts adjusted for various confounders in addition to a pooled analysis (not adjusted). They concluded that there was inconsistency in the included studies regarding thyroid cancer-related mortality (six adjusted studies included) and recurrence. Besides, they suggested beneficial effects of RAI remnant ablation in loco-regional recurrence (relative risk: 0.31; 95% CI: 0.2-0.49) and distant metastases (absolute decrease in risk: 3%; 95% CI: -0.01 to 0.04). In our meta-analysis, six of seven recent studies, of which five were adjusted [3,12-15], examined survival rate. Our results are in agreement with Sawka et al.’s results regarding the benefits of RAI use in remnant ablation. However, the present analysis included both low- and intermediate-risk DTC in contrast to the study by Sawaki et al. who assessed low-risk DTC. No doubt, the use of RAI in low-risk DTC might not overweigh the unwanted effects. The controversy floor is regarding the intermediate-risk group that we assessed. Therefore, our results need consideration due to the existing controversy and the lack of recent meta-analyses touching the same. About the recurrence, our analysis included only one study (the study by Hurtado-López et al. [11]) that we pooled among the studies assessing the survival rate. The aforementioned study was included despite investigating local recurrence in low-risk papillary thyroid carcinoma due to its importance (observational, longitudinal, and analytic of 10 years follow-up). The study showed no significant differences regarding multifocality, bilateral disease, and extracapsular invasion. Hurtado-López et al.’s [11] survey is ongoing because the updated American Joint Committee on Cancer (AJCC/TNM) staging system seems to be inadequate for predicting very low or low risk of survival expected in patients with DTC in stages I and II [17]. Also, the discrepancy in the shaded American Thyroid Association Risk Stratification System (ATA-RSS) regarding the definition of low and intermediate-risk DTC certainly affected the studies included in the current meta-analysis [18]. Sacks et al. [19] published a review based on high-quality studies in 2010 and found no survival benefit of RAI in low-risk patients in contradiction to high-risk patients; the evidence regarding the recurrence rate was mixed. Similarly, Zaman et al. [20] in their narrative review found high proponents of not using RAI in low-risk patients and its undisputed beneficial effects in those at a higher risk, leaving a grey area in-between. Our review showed no differences in patients at low/intermediate-risk DTC when RAI is used. However, the small number of the included studies, the heterogeneity observed, and the fact that we included studies assessing both recurrence and survival rate besides the observational studies analyzed prevented us to conclude and inform the surgeon’s community. The controversy since the era of Seidlin et al. Mazzaferri and Young, and Hay et al. [21-23] is still there due to the difficulty in conducting randomized controlled trials.

## Conclusions

No differences were found in patients with low/intermediate-risk DTC regarding locoregional recurrence and survival rate between TT followed by RAI and lobectomy alone. However, the current data were limited by the observational studies included, the pooling of both recurrence and survival rate, and the significant heterogeneity observed. The ongoing randomized controlled trials are awaited to resolve the issue.
